# Down syndrome with Alzheimer's disease brains have increased iron and associated lipid peroxidation consistent with ferroptosis

**DOI:** 10.1002/alz70855_105930

**Published:** 2025-12-24

**Authors:** Max A Thorwald, Jose A Godoy‐Lugo, Marc Vermulst, Henry Jay Forman, Elizabeth Head, Caleb E Finch

**Affiliations:** ^1^ USC Leonard Davis School of Gerontology, Los Angeles, CA, USA; ^2^ University of Southern California, Los Angeles, CA, USA; ^3^ University of California Merced, Merced, CA, USA; ^4^ University of California, Irvine, Irvine, CA, USA; ^5^ USC Dornsife College of Letters, Arts, and Sciences, Los Angeles, CA, USA

## Abstract

**Background:**

Increased brain iron is associated with sporadic Alzheimer Disease (AD) and Down Syndrome with AD (DSAD), which may involve iron from cerebral microhemorrhages (MBs). The prevalence of MBs is higher in DSAD, possibly from the triplication of the amyloid precursor protein on chromosome 21. Increased MB iron could cause oxidative damage through Fenton chemistry and subsequent lipid peroxidation. We hypothesize that iron and APP are intrinsically linked, and that triplication of APP would result in more tissue iron and lipid peroxidation than observed in sporadic AD.

**Method:**

Prefrontal cortex and cerebellum of cognitively normal, AD, and DSAD(*n* = 8/group) were examined for iron metabolism, antioxidant response, and amyloid peptides by immunoblot, inductively coupled mass spectrometry, and enzymatic assay.

**Result:**

Iron was 2‐fold higher in DSAD. Iron storage proteins and lipid peroxidation were increased in prefrontal cortex, but not in the cerebellum. The glutathione synthesis protein GCLM was decreased by 50% in both AD and DSAD. Activity of lipid raft GPx4, responsible for membrane repair, was decreased by at least 30% in AD and DSAD. These decreases in GPx4 activity were paralleled by reduced α‐secretase activity while β‐secretase activity increased.

**Conclusion:**

DSAD shows greater lipid peroxidation than AD consistent with greater MBs and iron load. DSAD also shares similar and more pronounced features of AD such as decreased protein levels of critical GSH producing enzyme GCLM and other protective mechanisms against lipid peroxidation. The extensive increase of iron and lipid peroxidation suggests their linkage to APP gene dosage. The impairment of these key mechanisms asserts ferroptosis as a key feature during AD.

